# Synthesis of Sulfonated Poly(Arylene Ether Sulfone)s Containing Aliphatic Moieties for Effective Membrane Electrode Assembly Fabrication by Low-Temperature Decal Transfer Methods

**DOI:** 10.3390/polym13111713

**Published:** 2021-05-24

**Authors:** Jieun Choi, Minkyu Kyeong, Minsung Kim, Sang-Soo Lee, Bora Seo, Hyun Seo Park, Hee-Young Park, Dirk Henkensmeier, So Young Lee, Hyoung-Juhn Kim

**Affiliations:** 1 Center for Hydrogen and Fuel Cell Research, Korea Institute of Science and Technology (KIST), Seoul 02792, Korea; t14896@kist.re.kr (J.C.); mkyeong@kist.re.kr (M.K.); brseo@kist.re.kr (B.S.); hspark@kist.re.kr (H.S.P.); parkhy@kist.re.kr (H.-Y.P.); henkensmeier@kist.re.kr (D.H.); 2 Soft Hybrid Materials Research Center, Korea Institute of Science and Technology, Seoul 02792, Korea; minsung@kist.re.kr (M.K.); s-slee@kist.re.kr (S.-S.L.)

**Keywords:** decal transfer method, glass transition temperature (T_g_), membrane electrode assembly (MEA), proton exchange membrane fuel cell (PEMFC), sulfonated poly(arylene ether sulfone)

## Abstract

The purpose of this study was to investigate the effect of the aliphatic moiety in the sulfonated poly(arylene ether sulfone) (SPAES) backbone. A new monomer (4,4’-dihydroxy-1,6-diphenoxyhexane) was synthesized and polymerized with other monomers to obtain partially alkylated SPAESs. According to differential scanning calorimetry analysis, the glass transition temperature (T_g_) of these polymers ranged from 85 to 90 °C, which is 100 °C lower than that of the fully aromatic SPAES. Due to the low T_g_ values obtained for the partially alkylated SPAESs, it was possible to prepare a hydrocarbon electrolyte membrane-based membrane electrode assembly (MEA) with Nafion^®^ binder in the electrode through the use of a decal transfer method, which is the most commercially suitable system to obtain an MEA of proton exchange membrane fuel cells (PEMFCs). A single cell prepared using this partially alkylated SPAES as an electrolyte membrane exhibited a peak power density of 539 mW cm^−2^.

## 1. Introduction

The proton exchange membrane fuel cell (PEMFC) is an electrochemical conversion technology that produces electricity and water from hydrogen and oxygen. The PEMFC is significant power generation technology in terms of the low-carbon economy, because these devices can complement the unstable supply of various energy sources. Indeed, the operation of PEMFCs could act as power sources for transportation, home, and industry, and research continues to increase in academia and industry to improve their associated performances and safety, and to lower the device costs [1]. Among the various components that make up a PEMFC, the most important is the membrane electrode assembly (MEA). An MEA consists of a polymer electrolyte membrane, an anode, and a cathode, and ultimately determines the overall performance of the PEMFC. Currently, poly(perfluorosulfonic acid)-type membranes, such as Nafion^®^, are commonly used as electrolyte membranes in PEMFCs. However, these membranes are expensive because of their complex manufacturing processes. As a result, significant research and development has focused on hydrocarbon-type proton exchange membranes with high conductivities and excellent mechanical and chemical stabilities [2,3,4].

The MEAs for PEMFCs are largely prepared via the catalyst-coated membrane (CCM) method [5]. The CCM method refers to all methods of applying catalyst layers (or electrodes) to an electrolyte membrane. Several CCM methods exist, including decal transfer, direct spraying, pulse spray swirling, ultrasonic spray deposition, and ink jet printing. Among them, the decal transfer method is considered to be the most commercially viable [6]. In the decal transfer process, a catalyst layer composed of a Pt/C catalyst and a polymer electrolyte binder is applied to an inert polymer substrate, such as Teflon or polyimide film, and then transferred to both sides of an electrolyte membrane by hot pressing. The hot-pressing step is performed at a temperature higher than the glass transition temperature (T_g_) of the electrolyte membrane and binder, and at this temperature, physical bonding between the electrolyte membrane and the binder is formed, thereby resulting in the manufacture of a stable MEA.

As mentioned above, the decal transfer method is advantageous in that the obtained MEA exhibits excellent interface characteristics, thereby rendering the continuous process more suitable for mass production [7]. The majority of studies carried out into decal process have been conducted at 120–130 °C for Nafion^®^-type membranes and binders, which have relatively low glass transition temperatures [8]. General hydrocarbon electrolyte membranes, which are superior in price to perfluorinated membranes, often exhibit a T_g_ above 200 °C, and therefore when manufacturing an MEA using a hydrocarbon electrolyte membrane, the decal transfer process must be performed at a temperature of ≥200 °C. When the Nafion^®^ electrolyte is used as a binder for hydrocarbon electrolyte membrane-based MEA manufacture, the decal transfer process cannot be performed by raising the temperature due to the decomposition of Nafion^®^ at these temperatures. The decal temperature is therefore selected by considering the glass transition and decomposition temperatures of the polymer [8]. In hot-pressing conditions, the crystallinity of the membrane increases and the electrode morphology can undergo deterioration, ultimately resulting in reduced proton conductivity and a smaller electrochemically active surface area (EAS), respectively [9]. Recently, a hydrocarbon binder was developed to replace the Nafion^®^ binder, and research is ongoing in this area. However, it should be noted that all MEAs were manufactured using the direct spraying method [10,11,12].

**Scheme 1:**
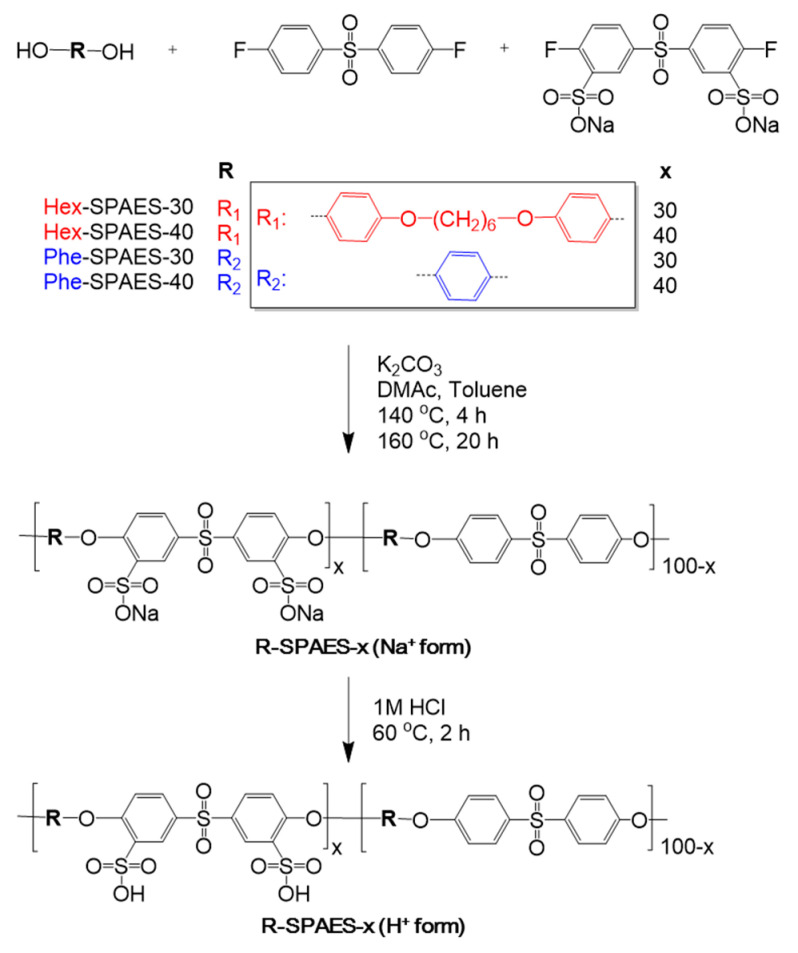
Synthetic route to Hex-SPAES-x bearing hexyl aliphatic chains and Phe-SPAES-x containing no aliphatic chains (“Hex” and “Phe” refer to the “hexyl” and “phenyl”, respectively; “x” represents the percentage of repeating units containing sulfonic acid).

Our interest therefore lies in the synthesis of a novel hydrocarbon electrolyte, namely, the partially alkylated sulfonated poly(arylene ether sulfone) (SPAES), Hex-SPAES (Scheme 1). This electrolyte exhibits a significantly lower T_g_ than the pristine SPAES, Phe-SPAES. Although some research has been carried out into modification of the T_g_ value for proton-exchange membrane polymers through copolymerization and blending [13,14,15], regulation of the T_g_ of a polymer with the purpose of optimizing the decal process for MEA fabrication is yet to be reported. By controlling the T_g_ of the SPAES membrane material, 98–100% transfer of the catalytic layer from the inert substrate to the electrolyte membrane is possible during hot pressing at 120 °C. This can address the issues related to electrolyte deterioration and incomplete transfer in the existing decal process. In addition, the introduction of flexible aliphatic chains is known to enhance the flexibility of the whole polymer main chain and facilitate the formation of ionic conduction channels by separating the hydrophilic/hydrophobic phases [16,17].

## 2. Materials and Methods

### 2.1. Materials

Hydroquinone (≥99%), 1,6-dibromoalkane (96%), bis(4-fluorophenyl) sulfone (99%), potassium hydroxide (90%), potassium carbonate (≥99%), sodium dithionite (99%), sodium chloride (≥99%), sodium hydroxide (98%), lead acetate trihydrate (≥99%), phenolphthalein (99%), petroleum ether (≥95%), anhydrous *N*-methyl-2-pyrrolidone (99.5%), and anhydrous toluene (99.8%) were purchased from Sigma-Aldrich, Inc. (Saint Louis, MO, USA) and used without additional purification. Bis(4-fluoro-3-sulfophenyl)sulfone disodium salt (≥99%) was purchased from Yanjin Technology Co., Ltd. (Tianjin, China). Sulfuric acid solution (5 N), ethanol (99.5%), and isopropanol (99.5%) were purchased from Daejung Co., Ltd. (Siheung, Korea). Prior to synthesis, all monomers and polymers were dried overnight in a vacuum oven at 60 °C. For the single-cell test, a 40 wt.% Pt/C electrocatalyst was purchased from Tanaka Kikinzoku Kogyo Co., Ltd. (Tokyo, Japan). The 5 wt.% Nafion^®^ ionomer solution and Kapton^®^ polyimide films were from DuPont Inc. (Wilmington, DE, USA), and Sigracet^®^ 39BC gas diffusion layers were from SGL Carbon Co. Ltd. (Wiesbaden, Germany).

### 2.2. Characterization and Methods

The ^1^H NMR spectra were obtained using a 400 MHz AVANCE-III spectrometer (Bruker, Billerica, MA, USA). The synthesized products were dissolved in deuterated dimethyl sulfoxide, and one drop of deuterated trifluoroacetic acid was added to each NMR samples to ensure that the water peak did not overlap with the sample signals. The molecular weights and polydispersity indices of the polymers were determined by gel permeation chromatography method using a Waters 1515 HPLC system (Waters Co., Ltd., Milford, MA, USA) equipped with a Waters 2414 refractive index detector. Two kinds of columns, namely, Waters Styragel^®^ HR 3 and HR 4, were used. The analysis was conducted at 40 °C and with a flow rate of 1.0 mL min^−1^ using 0.05 M LiBr in NMP as the eluent. Thermal gravimetric analysis (TGA) was performed using Q50 apparatus (TA Instruments, New Castle, DE, USA) and by heating the samples to 900 °C at a heating rate of 10 °C min^−1^ under a nitrogen atmosphere. Differential scanning calorimetry (DSC) measurements were carried out using a DSC 7 instrument (PerkinElmer, Inc., Waltham, MA, USA) under a nitrogen atmosphere. After isothermal holding at 30 °C for 3 min, each sample was heated to 350 °C at a heating rate of 10 °C min^−1^. After maintaining at this temperature for a further 3 min, the sample was cooled to 30 °C at a cooling rate of 10 °C min^−1^. This cycle was repeated, and the glass transition temperatures of the polymers were determined from a second non-isothermal scan from 30 to 350 °C. To observe phase separation, the acid forms of the membranes were treated with a 1 M aqueous lead acetate solution for 24 h, rinsed with deionized water several times, and dried in a vacuum oven for 24 h. The membranes were embedded in epoxy resins, cross-sectioned to 90 nm with an ultramicrotome system, and examined using a Tecnai G2 F20 transmission electron microscope (FEI Co., Hillsboro, OR, USA). The cross-sectional image of MEA was obtained using FEI-SEM Inspect F50 (FEI Co., Hillsboro, OR, USA) at an acceleration voltage of 10 kV.

The theoretical ion exchange capacity (IEC) values were determined from the molar ratio of monomers containing sulfonic acid groups. More specifically, the IEC value was calculated using Equation (1), where DS_Theoretical_ is the degree of sulfonation, and MW_repeat unit_ is the molecular weight of the polymer repeating unit.

IEC_Theoretical_ = (2 equivalents × DS_Theoretical_)/MW_repeat unit_ (1)

The titrated values of the membrane IECs were measured according to the classical titration method using phenolphthalein as an indicator [18]. After weighing the dried membrane, it was immersed in a 2 M sodium chloride solution for 48 h at 20 to 25 °C. The released protons within the solution were neutralized by the addition of a 0.1 N solution of sodium hydroxide. The IEC values were calculated from the titration results using Equation (2), where *V*_NaOH_ is the added volume of sodium hydroxide, *M*_NaOH_ is the molarity of the added sodium hydroxide, and *W*_dry_ is the weight of the dry membrane sample.

IEC_Titrated_ = (*V*_NaOH_ × *M*_NaOH_)/*W*_dry_ (2)

The water uptake results were calculated from Equation (3). The weight of the dry sample, *W*_dry_, was measured after drying the sample for 12 h in a 60 °C oven and subsequent cooling at 25 °C, while *W*_wet_ was measured after soaking the sample in DI water at 30 °C for 24 h and removing any remaining water on the surface of the sample. The dimension stability was also measured using a similar method and was calculated according to Equation (4).

Water uptake (%) = 100 × (*W*_wet_ − *W*_dry_)/*W*_dry_ (3)

Dimension stability (%) = 100 × (*l*_wet_ – *l*_dry_)/*l*_dry_ (4)

where *l*_dry_ and *l*_wet_ are the lengths of the dry and wet membranes, respectively.

To obtain values of conductivity, membrane samples were prepared in rectangular shapes (1 cm × 4 cm × 40 µm). After wetting in deionized water for 24 h, the sample was inserted in a four-probe cell manufactured for impedance measurements. The impedance was measured under conditions of varying temperature (30 °C, 40 °C, 60 °C, and 80 °C) and a relative humidity of 100% with the assembled conductivity cell placed in the chamber. Electrochemical impedance spectroscopy was carried out with a potentiostat (Biologics, SP-300) by applying a sinusoidal current signal with an amplitude of 5 μA, while the signal frequency was scanned from 1000 Hz to 7 MHz. The values of conductivity (*σ*) were calculated from the bulk resistance (*R*), the cross-sectional area (*A*) of the membrane sample, and the distance between the reference electrodes in the conductivity cell (*D*) using Equation (5) [19].

*σ* = *D*/(*R* × *A*) (5)

The mechanical properties of the polymer membranes were measured using a QC-508E instrument (Cometech Co., Ltd., Taichung, Taiwan) after drying at 60 °C in an oven and subsequent cooling. Rectangular samples with test areas of 1 cm (*l*_0_) × 1 cm (*w*_0_) × 40 µm (*d*_0_) were stretched at a rate of 0.5 mm min^−1^ at room temperature. The tensile strength and the percentage elongation were obtained from Equations (6) and (7), respectively. The Young’s modulus was determined from the initial slope of the stress–strain curve. All values were the averages determined using three repeat measurements.

Tensile strength = *F*/(*w*_0_ × *d*_0_) (6)

Percent elongation (%) = 100 × (*l* − *l*_0_)/*l*_0_ (7)

where *F* is the maximum load and *l* is the final length at point of fracture.

### 2.3. Synthesis of Sulfonated Poly(Arylene Ether Sulfone)s with Hexyl Aliphatic Chains (Hex-SPAES-30 Salt Form)

Sulfonated poly(arylene ether sulfone)s with hexyl aliphatic chains were synthesized by condensation polymerization in a 250 mL four-neck round bottom flask equipped with a mechanical stirrer, a Dean–Stark trap, a condenser, and an Ar-inlet adapter. The flask was charged with 4,4′-dihydroxy-1,6-diphenoxyhexane (3.0542 g, 10 mmol) [20], bis(4-fluoro-3-sulfophenyl)sulfone disodium salt (1.3889 g, 3 mmol), bis(4-fluorophenyl) sulfone (1.7977 g, 7 mmol), and potassium carbonate (2.8206 g, 20 mmol). Subsequently, anhydrous NMP (35.9 mL) and anhydrous toluene (18.0 mL) were added. The reaction mixture was heated at reflux at 140 °C for 4 h, and an azeotropic mixture of toluene and water was collected and removed using a Dean–Stark trap. After this time, the reaction temperature was increased to 170 °C, and the reaction was allowed to continue at that temperature for 20 h. The resulting viscous solution was poured into isopropanol (1 L) and washed several times with deionized water. The obtained polymer was filtered and dried at 60 °C under vacuum for 24 h (yield: 89.4%). This polymerization procedure was also applied for the preparation of Hex-SPAES-40 (yield: 89.0%). Phe-SPAES-30 and Phe-SPAES-40 were also synthesized according to this procedure using 4,4′-dihydroxydiphenyl ether instead of 4,4ʹ-dihydroxy-1,6-diphenoxyhexane (yields: 98.0 and 97.7%, respectively).

### 2.4. Membrane Preparation and Counter Ion Exchange from the Sodium Form to the Proton Form

Hex-SPAES-30 sodium form (1 g) was dissolved in NMP (19 g) to prepare a 5 wt.% solution. This solution was filtered using a 0.45 μm PTFE syringe filter and then cast onto glass dishes. After vacuum drying at 80 °C for 48 h, free-standing 40 μm thick membranes were obtained. To convert the polymer Na^+^ form into the H^+^ form, the membranes were immersed in a 1 M sulfuric acid solution at 60 °C for 2 h. After this time, the membranes were washed with DI water several times. The other membranes of a series of polymers were prepared in the same manner.

### 2.5. Decal Transfer and MEA Fabrication

To prepare the electrode slurry, a 40 wt.% Pt/C catalyst (1 g) was placed into a vial, to which distilled water (1.2 g) and the 5 wt.% Nafion^®^ ionomer dispersion solution (1.2 g) were added, along with isopropanol (1.2 g), under a nitrogen atmosphere. The resulting solution was stirred for 30 min, homogenized at 20 kHz for 5 min, then stirred for a further 24 h. The obtained catalytic slurry was cast onto the Kapton^®^ polyimide film, and the platinum catalyst was adjusted to 0.4 mg cm^−2^ using a doctor blade. The catalytic layer coated on the polyimide film was cut to 5 cm × 5 cm and then placed on both sides of the polymer electrolyte membrane to achieve attachment of the catalytic layer. A membrane electrode assembly (MEA) was manufactured by pressing at 120 °C for 5 min at a pressure of 100 kgf cm^−2^. The transfer rate was measured from the weight of the remaining catalytic layer on top of the polyimide film that was removed from the manufactured MEA.

### 2.6. Fuel Cell Tests of Membrane Electrode Assemblies (MEAs)

The single cell was assembled by placing the MEA between the gas diffusion layers and using graphite bipolar plates with a serpentine-type flow channel. The single-cell performance results of the MEAs were acquired from an ESL-300Z electronic load (E.L.P. Tek., Gunpo, Korea) and a fuel cell test station system (CNL Energy Co., Ltd., Seoul, Korea) including mass flow controllers, temperature control systems, and fuel cell control software. Humidified hydrogen (0.4 L min^−1^) and air (1.2 L min^−1^) were fed to the anode and cathode, respectively. After activation at a constant voltage of 0.4 V at 60 °C for 20 h, polarization curves were obtained at 60 °C and 95% relative humidity. Electrochemical impedance spectroscopy (EIS) was conducted using an HCP-803 potentiostat (BioLogic Science Instruments, France) equipped with EC-Labs software for monitoring the ohmic resistance and the charge transfer resistance of the single cells at 0.85 V.

## 3. Results and Discussion

### 3.1. Chemical Structure Analysis of the Synthetic Polymers

**Figure 1:**
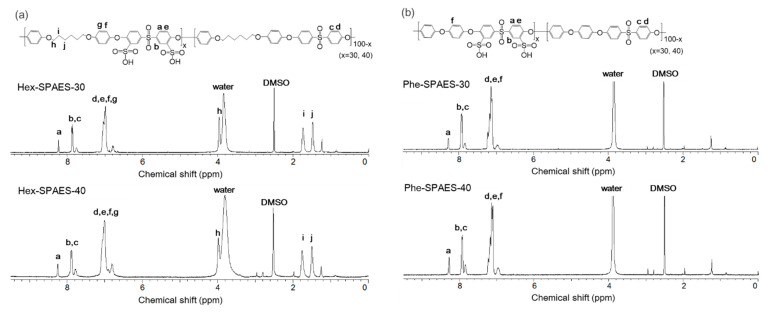
The ^1^H NMR (400 MHz; DMSO-*d*_6_;Me_4_Si) spectra of the synthetic polymer membranes: (**a**) Hex-SPAES-x bearing hexyl aliphatic chains; (**b**) Phe-SPAES-x containing no aliphatic chains (“Hex” and “Phe” refer to the “hexyl” and “phenyl”, respectively; “x” represents the percentage of repeating units containing sulfonic acid).

The ^1^H NMR spectra confirmed the successful preparation of SPAESs, both with and without hexyl aliphatic chains (Figure 1). In the case of Hex-SPAES, the peaks attributed to the hydrogen atoms in the aliphatic chains were observed to appear at δ 1.35–1.57, 1.57–1.88, and 3.87–4.08 ppm, and the peaks corresponding to the aromatic hydrogen atoms appeared at δ 6.61–7.34, 7.72–7.95, and 8.06–8.36 ppm.

Table 1 lists the polymeric properties of the Hex-SPAES and Phe-SPAES membranes. More specifically, the M_n_ values of Hex-SPAES and Phe-SPAES were in the ranges of 24,000–32,000 and 42,000–55,000, respectively, while the degrees of polymerization of the Hex-SPAESs containing hexyl aliphatic chains were lower than those of Phe-SPAESs without the aliphatic chains.

### 3.2. Effects of the Aliphatic Chain Contents in Polymers on the Thermal Properties

**Figure 2:**
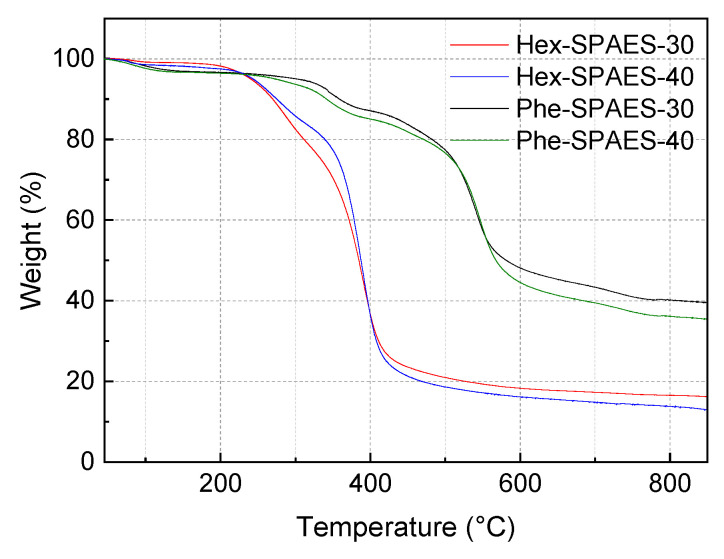
TGA thermograms of the synthetic polymer membranes.

Figure 2 shows the thermogravimetric analysis (TGA) results obtained for the synthetic polymers. The weight loss below 170 °C indicated evaporation of the residual moisture, while the sulfonic acid groups underwent decomposition between 200 and 400 °C. Finally, above 350 °C, breakdown of the polymer backbone took place.

**Figure 3:**
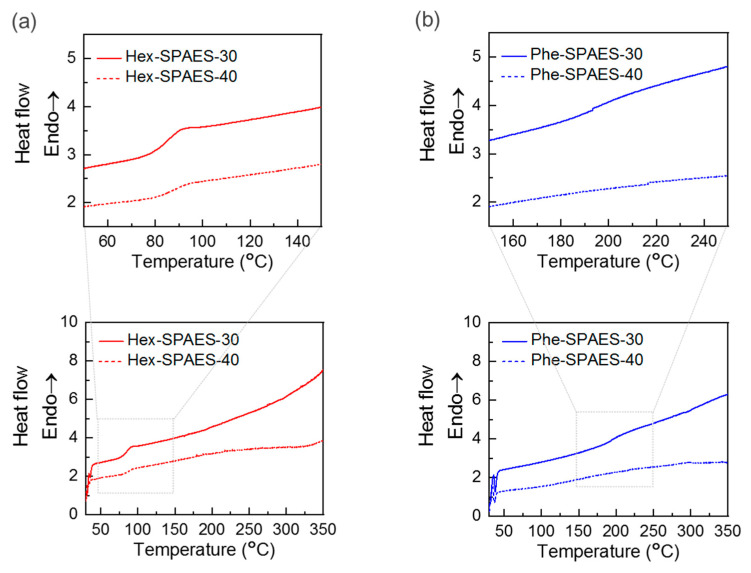
DSC thermograms of (**a**) Hex-SPAES-x bearing hexyl aliphatic chains; (**b**) Phe-SPAES-x containing no aliphatic chains, as recorded during the second scan.

The differential scanning calorimetry (DSC) results for determination of the T_g_ values of the polymers are shown in Figure 3. As indicated, the T_g_ values of Hex-SPAES-30 and Hex-SPAES-40 containing the hexyl aliphatic chain were confirmed to be 85.33 and 87.52 °C, respectively (Figure 3a), while those of Phe-SPAES-30 and Phe-SPAES-40, which contained no aliphatic chains, were 193.29 and 216.51 °C, respectively (Figure 3b). As a result, it can be seen that the T_g_ of the polymer electrolyte could be remarkably reduced by the introduction of the alkyl moiety.

There are many factors that affect the glass transition temperature of a polymer, such as the thermal properties of the components of the polymer chains [21], the molecular weight [22], and the type of chain connected [23]. Even if the glass transition temperature of the polymer is affected by molecular weight, even poly(arylene ether sulfone) with an M_n_ of less than 7000 rarely has a T_g_ of less than 150 °C [23]. Therefore, the T_g_ near 90 °C of the Hex-SPAES-30 and Hex-SPAES-40 cannot be due to a relatively low molecular weight and are due to the aliphatic structural components in the polymer. As reported in previously reported studies [24], the T_g_ of the polymer with a high degree of sulfonation shifts slightly toward higher temperatures. In the DSC results of Phe-SPAESs, it can also be seen that the T_g_ values are less clearly expressed because Phe-SPAESs are phenyl-rich rigid polymers.

### 3.3. Mechanical Properties of the Synthetic Polymeric Membranes

The ion exchange capacities (IEC) of the SPAESs were in the range of 1.1–1.7 meq g^−1^, and as expected, the IEC tended to increase with a higher degree of sulfonation. Similar trends were observed for the water uptake, dimensional stability, and conductivity (Table 2).

The conductivities of Hex-SPAES-30 and Phe-SPAES-30 were lower than that of Nafion 212. On the other hand, the conductivities of Hex-SPAES-40 and Phe-SPAES-40 were higher than that of the Nafion 212. IEC and the morphology of polymers are major factors in the ionic conductivity of polymer electrolyte membranes [25]. If the IEC values are similar, the conductivity of the Nafion 212 is higher than that of the synthesized SPAES membranes. Fluorinated groups of Nafion have higher electronegativity than the hydrogen of hydrocarbon-based membranes; therefore, it is known that Nafion is more conductive than general hydrocarbon-based membranes [26]. Hex-SPAES-40 showed a higher conductivity value, even though it had a lower IEC value than Phe-SPAES-40. Alkyl moieties in the backbone appear to have affected the morphology of the membrane [16]. It was shown that the conductivity of hydrocarbon-based membranes can be increased through the method of introducing alkyl chain moieties.

With the exception of elongation, the mechanical properties of Hex-SPAES and Phe-SPAES were similar (Table 3). Hex-SPAES exhibited approximately 30% greater elongation than Phe-SPAES; therefore, the introduction of the alkyl moiety was considered to play a role in increasing the toughness of the polymer. This is expected to be beneficial in terms of increasing its utilization as a polymer electrolyte membrane.

### 3.4. Morphology of the Synthetic Polymeric Membranes

There are many research papers that consider the degree of hydrophilic–hydrophobic phase separation in TEM images with respect to ion conduction channels [16,26]. This is because when the moisture content of the polymer electrolyte membrane increases, adjacent hydrophilic clusters connect to each other to form ion-conducting channels that promote ion diffusion [26]. Previously, Zhao et al. reported that the presence of a flexible aliphatic chain in the rigid aromatic polymer backbone increases the mobility of the polymer chain and contributes to the formation of a larger ion-conducting channel [16].

**Figure 4:**
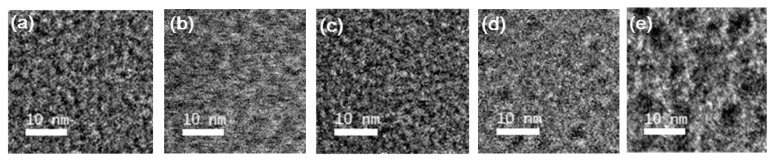
TEM images of the (**a**) Hex-SPAES-30; (**b**) Hex-SPAES-40; (**c**) Phe-SPAES-30; (**d**) Phe-SPAES-40; and (**e**) Nafion 212 membranes.

We therefore investigated the size of the hydrophilic channel through transmission electron microscopy (TEM) imaging of the Hex-SPAES and Phe-SPAES membranes; however, no significant differences were observed (Figure 4). Upon increasing the degree of sulfonation from 30 to 40, the hydrophobic segment became less prominent, and the size of the hydrophilic channel grew. It is believed that this change may occur through variation in the length of the alkyl chain, as will be examined in future studies.

The degree of hydrophilic–hydrophobic phase separation and ionic cluster sizes of general hydrocarbon-based membranes are known to be smaller than that of Nafion. This is because, in the case of general hydrocarbon-based polymers, the steric hindrance of the backbone of hydrocarbon-based membranes is greater than that of Nafion, and the hydrogen of the hydrocarbon-based membrane has lower electronegativity than the fluorine of Nafion [26]. Both Hex-SPAES-40 and Phe-SPAES-40 are nonfluorinated SPAES, but steric hindrance of the backbone of Hex-SPAES-40 has been partially reduced by the introduction of hexyl chains. This is thought to be the reason why Hex-SPAES-40 has higher conductivity than Phe-SPAES-40, as shown in Table 2.

### 3.5. Proton-Exchange Membrane Fuel Cell Test of the Fabricated Membrane Electrode Assemblies (MEAs)

The MEAs were prepared using Hex-SPAES-30, Phe-SPAES-30, and the Nafion^®^ binder. Table 4 shows photographic images of the MEAs after the decal transfer process, and also provides the catalyst transfer yields. The use of Hex-SPAES membranes bearing aliphatic chains enabled the manufacture of MEAs via a decal process involving pressing of the catalytic layer at a pressure of 100 kgf cm^−2^ for 5 min at 120 °C. The catalyst layers were then transferred to the membranes with areas of 5 cm × 5 cm. The transfer rate (98–100%) was measured using the weight of the remaining catalytic layer on the top of the substate film, i.e., the polyimide film, following its removal from the manufactured MEA. In the case of the Phe-SPAES membranes containing no aliphatic chains, transfer was difficult under the same temperature and pressure conditions. Thus, during the decal process, a temperature close to the glass transition temperature of the polymer should be applied to increase the catalyst transfer yield and enhance interfacial contacts between the membrane and the catalyst layers [8].

**Figure 5:**
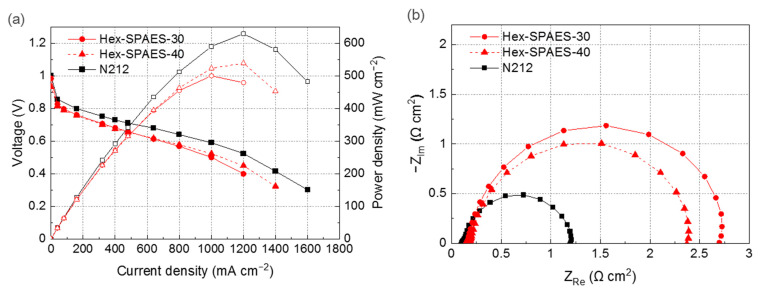
(**a**) Polarization curves and (**b**) EIS curves for the H_2_/Air PEMFCs employing Hex-SPAES-30, Hex-SPAES-40, and Nafion^®^ N212 membranes.

The single-cell test results for the Hex-SPAES-based MEAs and Nafion^®^ N212-based MEA are shown in Figure 5. The performance of the highly sulfonated Hex-SPAES-40 was superior to that of the Hex-SPAES-30. Overall, the MEAs manufactured from Hex-SPAES membranes exhibited approximately 79–86% of the maximum power density displayed by the MEA manufactured from a Nafion^®^ N212 membrane (Figure 5a). Analysis by electrochemical impedance spectroscopy (EIS) showed that the ohmic resistance and charge transfer resistance of the Hex-SPAES-based MEAs were larger than those of the N212-based MEA (Figure 5b). This seems to be due to the poor interfacial stability between the hydrocarbon polymer membranes and the perfluorinated Nafion^®^ polymer binder [29]. Indeed, this poor compatibility has been shown in the EIS, SEM, and peel strength test results of other literature [11,12]. We expect that it would also be possible to improve cell performance by applying hydrocarbon binders bearing the same backbone as the membrane [10,30,31].

### 3.6. Long-Term Test of the Fabricated Membrane Electrode Assemblies (MEAs)

**Figure 6:**
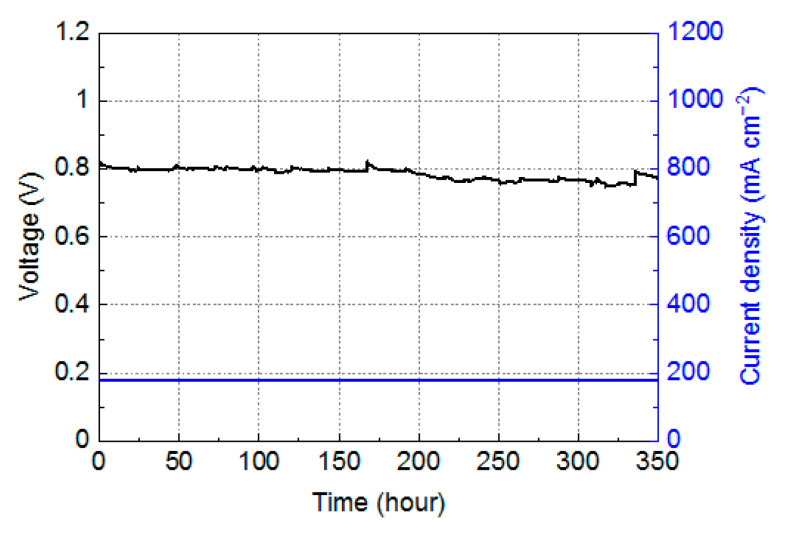
Plot of the cell voltage over time in constant-current operation of the MEA employing the Hex-SPAES-40 membrane.

**Figure 7:**
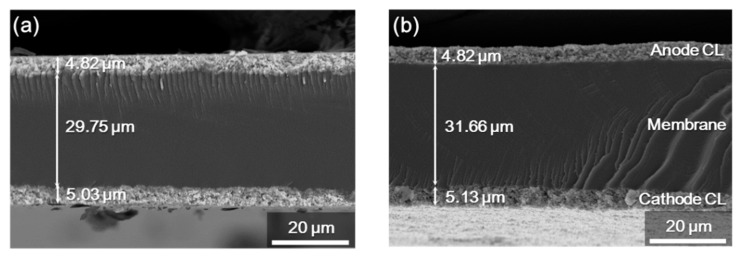
The SEM images showing the cross-section of Hex-SPAES-based MEA: (**a**) in pristine condition; (**b**) after constant-current operation for 350 h.

The long-term stability test with constant current mode were also performed. Figure 6 shows a plot of the cell voltage at 180 mA cm^−2^ over time. The 25 cm^2^ MEA with Hex-SPAES-40 membrane was operated at a constant current density of 180 mA cm^−2^ for 350 h. During fuel cell operation with constant current, the cell voltage remained stable for about 350 h. The average cell voltage was 0.786 V. When comparing the scanning electron microscopy (SEM) images of fresh and used MEA in the cross-section, the shape of the MEA was maintained even after operation for 350 h (Figure 7). There was no delamination of catalyst layers from the membrane and no change in thickness. Experiments for the degradation of the two types of membranes are still in progress, which will be covered in subsequent studies. Long-term single-cell tests of MEAs are being conducted to observe changes in the current density at constant voltage, changes in the open circuit voltage (OCV) state, and changes in I–V curves under repeated harsh operating procedures, where humidification and drying are alternated under different temperature and relative humidity operating conditions.

## 4. Conclusions

In conclusion, for the first time, sulfonated PAESs bearing alkyl moieties in the backbone were synthesized to produce polymer electrolytes with remarkably low T_g_ values. The polymers had a much lower T_g_ than pristine sulfonated PAESs. We produced MEAs with sulfonated PAES membranes containing alkyl chains in the polymer backbone by the decal transfer method. This was the first study to manufacture hydrocarbon-based MEAs using the common decal transfer method. These polymers exhibit excellent thermal and mechanical properties and are suitable for the manufacture of PEMFC MEAs. Currently, the performance of the prepared SPAES-based MEA is lower than that of MEAs manufactured using commercially available electrolyte membranes; however, it is expected that an MEA with increased performance will be obtained when an appropriate hydrocarbon electrolyte is synthesized as a binder.

## Figures and Tables

**Table 1:** Molecular weights of the synthetic polymer membranes.

Sample	M_n_^a^ (g mol^−1^)	PDI ^b^
Hex-SPAES-30	32,378	2.23
Hex-SPAES-40	23,999	1.74
Phe-SPAES-30	54,956	2.51
Phe-SPAES-40	42,401	2.26

^a^ Determined by gel permeation chromatography (GPC); ^b^ polydispersity index (M_w_/M_n_).

**Table 2:** IEC, water uptake, dimensional stability, and conductivity values for the synthetic polymer membranes.

Sample	IEC_Theoretical_^a^ (meq g^−1^)	IEC_Titrated_ (meq g^−1^)	Water Uptake (%)	Dimensional Stability (%)		Conductivity (S/cm)				Reference
				*x*–*y* Plane	*z*-Axis	30 °C	40 °C	60 °C	80 °C	
Hex-SPAES-30	1.1	1.1 ± 0.1	6.6 ± 0.9	4.4 ± 0.3	3.2 ± 1.9	0.05	0.08	0.10	0.11	
Hex-SPAES-40	1.4	1.4 ± 0.1	10.1 ± 3.2	4.6 ± 1.1	8.5 ± 1.2	0.15	0.18	0.22	0.29	
Phe-SPAES-30	1.3	1.3 ± 0.1	9.8 ± 3.0	2.2 ± 1.3	6.2 ± 1.9	0.04	0.05	0.07	0.09	
Phe-SPAES-40	1.7	1.4 ± 0.2	19.3 ± 5.5	5.0 ± 0.7	8.1 ± 2.1	0.07	0.13	0.19	0.27	
Nafion 212	1.0	-	20 ± 2	9.5 ± 1.9	12.7 ± 2.5	0.09	0.13 ^a^	0.16	0.18	[27,28]

^a^ Calculated from the chemical structures.

**Table 3:** Mechanical properties of the synthetic polymer membranes.

Sample	Yield Strength (MPa)	Tensile Strength (MPa)	Elongation (%)	Young’s Modulus (GPa)	Reference
Hex-SPAES-30	38.2 ± 2.7	41.9 ± 6.9	184.0 ± 37.7	0.55 ± 0.06	
Hex-SPAES-40	34.3 ± 4.0	41.1 ± 4.6	198.6 ± 8.6	0.51 ± 0.06	
Phe-SPAES-30	40.7 ± 1.4	41.7 ± 2.2	151.1 ± 18.1	0.68 ± 0.01	
Phe-SPAES-40	40.4 ± 0.9	41.0 ± 0.1	162.2 ± 28.6	0.61 ± 0.03	
Nafion 212	-	25.6 ± 1.4	365.2 ± 63.1	0.12 ± 0.01	[27]

**Table 4:** Photographic images of the membrane electrode assemblies after the decal transfer process, and catalyst transfer yields achieved using the Hex-SPAES-30 and Phe-SPAES-30 membranes.

Sample	Photographic Images After the Decal Transfer Process				Catalyst Transfer Yield (%)	
	Substrate		MEA			
	Anode	Cathode	Anode	Cathode	Anode	Cathode
Hex-SPAES-30	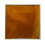	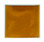	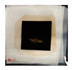	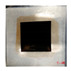	98	100
Phe-SPAES-30	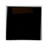	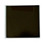	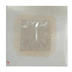	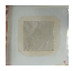	3	3

## Data Availability

The data presented in this study are available on request from the corresponding author.
